# Quantification of airborne fungal antigens by ELISA and comparison to molecular biological and classical methods

**DOI:** 10.1128/aem.00163-25

**Published:** 2025-07-16

**Authors:** C.-E. Pogner, M. Gorfer, M. Raulf, J. Strauss, I. Sander

**Affiliations:** 1 Bioresources, AIT Austrian Institute of Technology GmbH 31189, Tulln, Austria; 2 Institute of the Ruhr-University Bochum (IPA), Institute for Prevention and Occupational Medicine of the German Social Accident Insurance 14907 , Bochum, Germany; 3 Institute of Microbial Genetics, Department of Applied Genetics and Cell Biology, University of Natural Resources and Life Sciences (BOKU) 69516, Tulln, Austria; Royal Botanic Gardens, Surrey, United Kingdom

**Keywords:** bioaerosols, colony-forming units, qPCR, bioaerosol chamber, spore counts, immunoassay, filtration sampling

## Abstract

Airborne fungal spores are recognized for their human health impact, yet dose-response relationships remain undefined despite decades of bioaerosol sampling and analysis. In this article, we quantify immunologically active compounds, using six fungal-specific enzyme-linked immunosorbent assays (ELISAs) and compare them with cultivation, spore counting, and quantitative PCR (qPCR), using identical filter extract aliquots, various concentrations, single-organism aerosols, and mixes, in a controlled environment. Results showed high agreement between ELISAs and spore counting. By contrast, CFU enumeration and qPCR showed lower correlation with the other methods. Results of ELISAs showed high reproducibility of technical replicates and sampling duplicates, whereas CFU and qPCR results had high deviations. ELISA detection limits ranged from 10 to 100 spores/mL for larger spores (*Cladosporium herbarum*, *Aspergillus amstelodami*) and from 103 to 104 spores/mL for smaller spores (*Aspergillus amoenus*, *Aspergillus fumigatus*, *Penicillium chrysogenum*). The *Wallemia sebi* and the *Penicillium chrysogenum* ELISAs cross-reacted to *Aspergillus protuberus* or *Aspergillus amstelodami*, respectively. The spore dust production method influenced the spore germination rate and qPCR results significantly. By contrast, ELISA results remain unchanged, with the exception of the *W. sebi* ELISA. Quantitative spore counting was challenged by mixtures of species with morphologically similar spores from different fungal groups. In conclusion, the ELISA method is found suitable for fungal antigen quantification of air samples, provided the assays are sufficiently sensitive and specific, and germination evaluation is not required for risk assessment, leading to *C. herbarum* and *A. amstelodami* ELISA recommendations.

**IMPORTANCE:**

Bioaerosol detection and analysis is an ongoing field of research. Although various methodologies are used for collection and analysis, there is no single method available to close the knowledge gap between exposure and health impact. For airborne fungal material, the standard analysis method remains cultivation. With molecular technology advancing in the field, both methods can only show the exposure to living, cultivatable, or total fungal cells in the airborne environment. To close the gap between airborne concentrations and impact on the human body, recognition of the allergenic potential is necessary. Therefore, we evaluated six fungal-specific ELISAs to make them ready for application in field studies and compared them to cultivation, spore counting, and molecular genetic methods. We are confident that in the future antigen-recognizing methods like the tested ELISAs will enable moving from particle detection toward detection of the disease-causing agent.

## INTRODUCTION

Microbial airborne particles, like fungal spores and bacterial aggregates, can be inhaled and lead to impaired health in animals and humans. Frequent contact with high concentrations of these bioaerosols can lead to specific or nonspecific reactions of the immune system. These range from irritation and swelling of the nasal membrane (1), to allergies and airway irritation (2), and even to chronic lung disease (3). Particularly, repeated inhalation of fungal spores and other fungal components can lead to sensitization and the development of allergies. Severe cases can be diagnosed as exogenous allergic alveolitis or allergic asthma (4–6). On many occupational sites, a high concentration of bioaerosols and repeated exposure may lead to health effects for workers (7, 8).

Although the connection is already widely recognized, the link between exposure assessment and disease development, due to multiple influences on the various steps of assessment, is still not uncovered (9, 10). The first step in uncovering the connection is the measurement of exposure. But the reliable and comprehensive detection of bioaerosols is still a field of research, as no method can detect the entire spectrum of components and the total concentration (11).

There are diverse methods for detecting airborne fungal particles: cultivation of live propagules and microscopic detection belong to the oldest and frequently used methods, and DNA-based methods are on the rise due to the reduction in costs over the last decades.

Cultivation methods are still widely used and recognized as the standard in normative documents. However, there are two main issues with the standard approach. Only fungal species cultivable on standard media can be detected, and only unharmed live spores can be grown for detection. Furthermore, damaged spores as well as mycelium debris are important to detect, as they may also lead to adverse health effects, due to the presence of many allergens in the entire organism (12). Microscopic particle enumeration techniques alleviate the requirement for live spore material for cultivation. However, even using advanced image processing software, some taxa are very difficult to identify based on their optical features (13, 14). To overcome this limitation, advanced holographic imaging methods have been shown to provide sufficient resolution to distinguish single spores from aggregates and chains, thus overcoming the limitations of cultivation-dependent methods (15).

Modern non-cultivation methods, which offer a high resolution for differentiation of taxa, are based on DNA extraction and molecular fingerprinting, but for these methods, various steps of sample preparation are necessary after sampling, lowering the detection limit (16, 17).

Besides challenges in the differentiation of taxa and correct quantification over a wide range of concentrations, various fungal taxa have different allergenic potential of the spores (12). To be closer to the health effect in humans, detection methods based on the detection of antigens and allergens instead of the spore concentration were developed (18–21). As proteins are responsible components for sensitization and the development of allergies, these methods may provide a missing link between the concentration of airborne fungal propagules and their effect on human health.

The detection methods are based on immunological methods, enzyme-linked immunosorbent assays (ELISA), which are used for quantification of antigens and allergens. The Institute for Prevention and Occupational Medicine of the DGUV, Institute of the Ruhr-University Bochum (IPA), has developed six fungal-specific ELISAs for the detection of fungal antigens.

To validate the detection methods, we compared them with the standard cultivation method. In addition, newly obtained exposure assessment can only be related to existing data by comparison with existing quantification methods. In a previous study (22), some of the ELISAs, developed by the IPA, were already compared to cultivation and microscopic counting in field studies. Field studies provide a range of different samples and enable testing under real conditions. However, the production of replicates of similar samples for systematic comparisons is rarely possible.

To produce multiple samples with comparable amounts of bioaerosols, chamber studies are used (see summary in supplementary 11 [23–26]). These studies are very diverse, where aerosol generators of various types and aerosol chambers of different sizes and shapes are used; therefore, the comparability of different studies is limited. Furthermore, bioaerosols are produced from controlled starting material but various organisms, further reducing comparability between different studies. The starting material is usually a single organism or a mix of organisms, for example from media plates with fungal colonies. Tests with aerosol mixes are especially important, as aerosols of individual fungal species do not occur in the natural environment. Aerosol generators can produce various concentrations of aerosols, depending on the aerosolization methods used (23, 27). Bioaerosol chambers fulfil different purposes and can therefore be designed differently, with a chamber volume ranging from only half a liter to over 90 m³, with or without reuse of the particle flow, with or without uniform particle distribution (see supplementary table ST1 in references 11, 28).

In the study presented here, we produced bioaerosols from seven fungal species using two different bioaerosol generators connected to a 1.2 m³ bioaerosol chamber with equal particle distribution. The chamber was tested for producing homogeneous aerosols in the sampling area with multiple sampling points and over a long period of time (11, 25, 29). In this setup, we produced 222 filter samples loaded with various concentrations of fungal spores. Using the extracts of these filters, we compared the developed ELISAs with the standard cultivation technique as well as cultivation-independent microscopic spore counting and DNA quantification using qPCR.

## MATERIALS AND METHODS

### Organisms and cultivation

Seven different fungal species were used in the study presented here. From all, liquid spore suspensions were prepared and used in the described experiments. Species were used either as single organisms or as a mix of five fungi. The strains originated from in-house strain collections of the project partners (AIT, BOKU, and IFA) or were obtained from ATCC (Table 1). All strains were checked for their species assignment by sequencing of three phylogenetically informative DNA regions (ITS, β-Tubulin, and EF1α) and database comparison prior to use.

**TABLE 1:** List of used fungal species and strains as well as respective cultivation media^*a*^

Species	Risk level	Strain	Isolated from	Cultivation media	ITS/LSU	bTub	EF1α
*Aspergillus amoenus*	1	ATCC10072	Mammary gland	MEA		JN853939	
*Aspergillus protuberus*	1	V27.11	Samples from building restoration	MEA		PV642581	
*Aspergillus amstelodami*	1	RL586	Placenta porcine, veterinary laboratory	DG18		PV642580	
*Aspergillus fumigatus*	2	RL578	Bioaerosol sampling outdoor, compost	MEA		PV642579	
*Cladosporium herbarum*	1	D_D78	Bioaerosol sampling, indoor	MEA	PV620838	PV642578	PV642582
*Penicillium chrysogenum*	1	D_D53	Bioaerosol sampling, indoor	MEA		PV642577	
*Wallemia sebi*	1	MA3989	Bioaerosol sampling, indoor	MEA + 0.5% NaCl	PV620839		PV642583

^*a*^ Columns ITS/LSU, bTub, and EF1α show the accession numbers for analyzed markers. MEA, malt extract agar; DG18, dichloran 18% glycerol agar.

For spore production, biosafety level 1 (BSL-1) strains were cultivated on their respective media (Table 1) on petri dishes, and spores were harvested with a metal spatula after 10–14 days. For liquid aerosolization, the material was transferred to 1× phosphate-buffered saline (PBS) + 0.01% Tween 20 (PBST) buffer, vortexed for 3 minutes, and filtered through sterile glass wool (11). For each bioaerosol chamber experiment, the liquid spore suspension was prepared freshly. To produce dry spore material, harvested conidia were transferred into clean water (Arium pro, Satorius), vortexed, filtered, frozen with liquid nitrogen, and freeze-dried until powder state (Alpha 2-4 LSC, CHRIST) as described previously (30). The final spore powder was stored sealed at room temperature until use.

For biological safety reasons, the potentially harmful fungus *A. fumigatus* was cultivated in a BSL-2 facility on slant agar in 50 mL tubes sealed with parafilm. For harvesting, buffer was added directly to the tube, vortexed, and this suspension was filtered through sterile glass wool.

As aerosol experiments were conducted in a bioaerosol facility with biological safety level 1, work with living and potentially infectious *Aspergillus fumigatus* spores was not allowed. Therefore, the spore suspensions were inactivated by treatment with an antifungal agent with a final concentration of 2.5% for 12 hours (Baufan Bauchemie Leipzig GmbH; containing 2.49% 2-Octyl-2H-isothiazol-3-on). Preliminary tests confirmed efficient killing of *A. fumigatus* spores by this treatment and showed the same amount of antigen/spore for untreated and treated fungal spore extracts (Fig. S2).

The species *Aspergillus amoenus* and *Aspergillus protuberus* both belong to the clade and subclade *Aspergillus versicolor*, which was introduced in 2012 (31). Prior to that publication, *A. versicolor* was a species with documented genetic and phenotypic variation that did not resolve into clearly recognizable species. *A. versicolor* was recognized as an indicator for Sick Building Syndrome (32) and often reported for damp indoor environments (33, 34).

### Aerosol production

To produce aerosols in a controlled environment, two different aerosol generators were connected to the aerosol chamber (CCB3000, Palas GmbH). The liquid sparging aerosolizer (SLAG, CH Technologies) was used for liquid suspensions to produce low concentrations of fungal spores in the air. For high concentrations, the rotating brush generator (RBG1000, Palas GmbH) was used with the conidial spore powder. Both systems are compatible with the aerosol inlet of the aerosol chamber and have been used in previous studies in this combination (11, 17, 25, 29). With the use of both aerosol generators and five settings, it was possible to produce different concentrations of fungal spores in the chamber, for each of the BSL-1 test organisms (SLAG for two low concentrations, RBG for three high concentrations). For *A. fumigatus*, no dry spore powder was produced, as inactivation was only tested in liquid, and experiments with an active BSL-2 organism in the BSL-1 aerosol laboratories were not permitted.

To verify the concentration of particles in the test system and check the antigen content after aerosolization, three monitoring points were used in the system: (i) the starting material (powder or liquid suspension), (ii) the aerosol stream directly after the generator sampled into a gas wash bottle (11), and (iii) the particle concentration on the sampling position. The material of the first two monitoring points, as well as from the sampled filters (see below), was evaluated with four different analytical methods: microscopic spore counts, CFU counts, qPCR, and ELISA.

### Aerosol sampling and extraction

For each combination of fungal species and aerosolization setting, at least five sampling replicates were performed. Two identical sampling devices were used in parallel. The specific settings were different for each fungus, as the efficiency of aerosol production varied between species. The settings included the type of aerosol generator, the concentration of the source material, the settings of the aerosol generator, and the sampling time.

To evaluate the impact of aerosolization on the fungal spores, the airstream was collected into a gas washing bottle, filled with 40 mL PBST, directly after the aerosol generator at the start of each experimental setup. Sampling was conducted for 40 minutes for the liquid aerosolizer and for 2–5 minutes for the rotating brush generator (11, 30).

The air samples were collected on a Teflon filter (FALP0037, Merck Milipore), inserted into a GSP filter cassette, with a sampling cone appropriate for a 10 L/min airflow. Gilian 10i pumps were used to produce the airflow, which was verified with a rotameter on each sampling day. Sampling was performed for 10–90 minutes, depending on the experimental settings and spore concentration in the chamber, to enable the production of the various concentrations of spores on the filters.

After sampling, the filter holders and filters were removed from the sampling device and transferred into the GSP transport containers. In a sterile workbench, the filter holders were disassembled, and the filters were transferred into 15 mL tubes containing 5 mL PBST buffer. For the extraction of sampled conidia, the filters were vortexed for 1 minute at full speed and subsequently placed on a roller shaker for 30 minutes. These steps were repeated once. The extract was aliquoted and used for the four analytical methods.

### Detection methods

For the quantification of fungal material, four different methods were used. All these methods were applied to the source material, the liquid of the gas washing bottle, and all filter samples.

#### Spore counting

For spore counting, appropriate dilutions of the suspensions were counted under the microscope (Eclipse E200, Nikon) with a 400-fold magnification using a disposable counting chamber (Neubauer Improved C-Chip, Incyto), where 10 µL of liquid was applied. The concentration was calculated according to the manufacturer’s instructions. As only 0.9 µL of the liquid is covered by the counting grid (3 × 3 mm, 0.1 mm depth), this detection method has a lower limit of quantification of 5,000 particles/mL. All microscopic counting was performed by the same person.

#### Cultivation

For enumeration of colony-forming units, a suitable dilution (calculated from the spore concentration) was plated on three plates containing malt-extract agar and cultivated for 5–7 days. The colonies were counted, and the concentration calculated.

#### DNA extraction and qPCR

For quantification based on molecular detection, an aliquot (1 mL) of each sample was used. The samples were frozen at −80°C in a 96 deep well plate directly after production of the sample. For DNA extraction, the MagAttract PowerSoil Kit (Qiagen) was used with some modifications. For processing of the whole plate, 500 µL Power Bead buffer and 1.26 g of matrix Y (MP Bio) were added. Bead beating was performed at the highest speed (1,800 rpm) on a Fast Prep 96 bead beater (MP Bio) for 3 × 30 seconds. The plate was centrifuged for 10 minutes at 4,000 *× g*, and the supernatant was transferred into a 96-well plate of the kit. The subsequent steps were executed according to the manufacturer using a robotic system (Microlab Star, Hamilton).

For the qPCR, the DNA was diluted 1:10 and analyzed with different assays (specific or general, for primer details, see Table 2). For samples containing only one organism, the sensitive FungiQuant assay was used. For mixed samples, specific assays for *Aspergillus*, *Cladosporium*, *Penicillium,* and Basidiomycetes were applied. All assays target the SSU region (Small subunit ribosomal RNA) of the fungal cells (Table 2). To quantify the results, standards were used in each qPCR run. Standards were generated from each organism by replicating a long PCR fragment spanning nearly the entire SSU region and the ITS region (about 1,800 base pairs) using purified genomic DNA from appropriate pure fungal cultures as templates (Table 2). PCR product was purified using the QIAquick PCR Purification Kit (Qiagen) and the DNA concentration measured (Quant-iT dsDNA Assay Kit, High Sensitivity, Invitrogen). The copy numbers of the standards were calculated from the length of the product and the DNA concentration.

**TABLE 2:** List of primers and their respective targets

Target organism	Primer	Primer sequence	Reference
Fungi	FungiQuant-F	5′-GGRAAACTCACCAGGTCCAG-3′	(35)
	FungiQuant-R	5′-GSWCTATCCCCAKCACGA-3′	(35)
Basidiomycetes	Basid2R+	5′-TACCGTTGTAGTCTTAACAG-3′	(36)
	FungiQuant-R	5′-GSWCTATCCCCAKCACGA-3′	(35)
*Aspergillus*	ASP-F1	5′-TGCGATAACGAACGAGACCTCGG-3′	(17)
	ASP2	5′-ACCCCCCTGAGCCAGTCCG-3′	(37)
*Cladosporium*	CLA-F1	5′-TTCACTGGGCGTGTTG-3′	(17)
	CLA-R1	5′-GAACCACACGTCCTAT-3′	(17)
*Penicillium*	PEN-F1	5′-GAGAACAATTTAAATCCCTT-3′	(17)
	PEN-R1	5′-GGGTCATYATAGAATCC-3′	(17)
Primer for qPCR standard	ITS4	5′-TCCTCCGCTTATTGATATGC-3′	(38)
	NS31	5′-TTGGAGGGCAAGTCTGGTGCC-3′	(39)

All qPCRs were performed in four replicates with a final reaction volume of 5 µL in a 384-well plate (Biorad). The reaction mix consisted of 2.5 µL GoTaq-qPCR (Promega), 1 µL sample, 0.9 µL PCR-grade H_2_O, and 0.3 µL primer each from a 10 µM stock solution. The protocols were run on a CFX 384 (Biorad) using the following programs: FungiQuant—95°C for 5 minutes, 40 cycles of 95°C for 20 s, 54°C for 20 s, 72°C for 30 s; specific assays—95°C for 5 minutes, 40 cycles of 95°C for 20 s, 55°C for 20 s, 72°C for 25 s.; after the 40 cycles, a melting curve from 65°C to 95°C was produced.

#### Fungal-specific ELISA

All fungal-specific ELISAs were produced by the IPA in Bochum, Germany, and are based on polyclonal antibodies raised in rabbits against total fungal extracts of the respective species. In Table 3, the source of the immunogens, standards, and substrates, as well as the detection limits based on protein concentration of their standards, are presented. Antigen concentrations were calculated using the 4-parameter fit of the dose-response curve of a dilution series of the standard material and its protein concentration. The detection limit of each ELISA was the mean antigen concentration corresponding to a fixed value above the plate background and above the estimated minimum value of the 4-parameter curve fit function.

**TABLE 3:** Data for fungal-specific ELISAs developed by IPA in Germany

	Source of immunogen and standard	Substrate	Detection limit [ng/mL]	Publication
*Aspergillus fumigatus*	Greer (My3)	K-Blu (Neogen)	0.11	Unpublished
*Aspergillus amoenus* (clade *A. versicolor*)	Allergon (no. 1015)	ABTS (Sigma)	0.12	(21)
*Aspergillus amstelodami* (formerly: *Eurotium amstelodami*)	DMZ (strain 62629)	ABTS (Sigma)	0.013	(19)
*Cladosporium herbarum*	Immunogen: DMZ (strain 63422) Standard: Allergon (no 1034)^*a*^	ABTS (Sigma)	0.018	(20)
*Penicillium chrysogenum*	Allergon (no. 1090)	ABTS (Sigma)	0.08	(20)
*Wallemia sebi*	DMZ (strain 5329)	ABTS (Sigma)	0.2	(19)

^*a*^ For *Cladosporium herbarum*, a different source for the immunogen and the standard was used.

The *Aspergillus amoenus* material used for ELISA production was purchased as *Aspergillus versicolor* from Allergon (21). After the publication of the molecular relatedness in the clade *Aspergillus versicolor* in 2012 (31), the dry material used to establish the assay (21) was analyzed using β-Tubulin sequencing, which revealed that the material belongs to *Aspergillus amoenus*. For this sequencing, the same procedure as for checking the organisms for aerosol production was employed (see “Organisms and cultivation,” above).

The extraction of the antigens used for immunization and standards, as well as the components and the measurement of the sandwich ELISAs, were already published before (19, 20), except for the *Aspergillus fumigatus* assay.

Of all collected samples in the here-presented study, two aliquots (1 mL) were stored at −80°C and transferred frozen from the AIT to the IPA, for subsequent antigen quantification using the ELISAs. After thawing, two different methods for the preparation of samples were applied: mixing extraction and bead beating. For the mixing extraction, samples were first vortexed for 1 min and then incubated on a roller mixer for 30 min. For cell disruption by bead beating, a Precellys homogenizer, SK38, matrix was used. Three cycles of bead beating at 6,000 rpm for 20 s and pausing for 30 s were applied. The homogenizer was cooled to 4°C–6°C by means of a Cryolys cooling system. Both extracts were centrifuged for 15 min at 3,000 *× g*. The supernatants were subsequently used in the ELISA measurement.

The extracts were tested in a 1:2 serial dilution series to meet the measurement range. In the experiments for the characterization of the ELISAs with regard to their reactivity (see “Cross-reactivity of ELISA tested in liquid spore suspensions,” below), spore extracts were used in up to 18 serial dilutions. Otherwise, the extracts were tested in three serial dilutions and repeated until at least two values were within the measuring range.

### Statistics and calculations

From the raw data obtained with each detection method, the following derived values were calculated:


$$Antigen/spore\left[ng/spore\right]=\frac{Antigen\frac{ng}{ml}}{spore\;count\;spores/ml}$$

$$copy\;number/spore\left[cp/spore\right]=\frac{copy\;number/ml}{spore\;count/ml}$$

$$germination\;rate\left[gr\in \text{\textbackslash{}\% }\right]=\frac{colony\;forming\;units/ml}{spore\;count/ml}\times 100$$

#### Calculation of reactivity from ELISA

The spore extracts of six fungal species were measured with each ELISA. The relative reactivity was calculated according to the following formulas, where ELISA spec represents the target-specific result and ELISA the non-target result:


$$Reactivity\;based\;on\;protein=\frac{Antigen\;ELISA\frac{ng}{\mu gprotein}}{Antigen\;ELISA\;spec\frac{ng}{\mu gprotein}}$$

$$Reactivity\;based\;on\;spore\;count=\frac{Antigen\;ELISA\frac{pg}{1,000spores}}{Antigen\;ELISA\;spec\frac{pg}{1,000spores}}$$

#### Limit of detection of ELISA

The limit of detection (LoD) of each ELISA was calculated based on the protein concentration of the standard material (see “Fungal-specific ELISA,” above) (Table 3).

The limit of detection based on spore count (spore number/mL) of each sample was calculated as follows:


$$LoD\frac{spore\;number}{mL}=\frac{LoD\frac{ng}{mL}}{Antigen\frac{ng}{spore\;number}}$$

#### Variation coefficient of technical replicates

To evaluate the variation of spore counting, one suspension of *Penicillium chrysogenum* (7 × 10^5^ spores/mL) was counted 10 times by the same person who counted all other suspensions in the study presented here. The results show a mean concentration of 6.7 × 10^5^ spores/mL, a standard deviation of 2.6 × 10^4^ spores/mL, and a relative standard deviation of 3.73%.

For the calculation of the coefficient of variation (CV) for cultivation, the mean, standard deviation, and relative standard deviation (in percent) were calculated for each sample. The mean and percentiles of these relative standard deviations were calculated for all samples as well as for each organism individually.

For ELISA measurements, the mean and relative standard deviation were calculated from the concentrations of usually three, but at least two, values in the measuring range of the dilution series.

For qPCR, the CV was calculated in a similar way. The mean, standard deviation, and relative standard deviation were calculated from the four replicates after conversion to copy numbers. The mean, quantiles, and percentiles of these relative standard deviations were calculated for all samples as well as for each qPCR assay individually.

#### Variation of analysis and sampling pairs

To evaluate variations in the artificial setup, we analyzed the difference between the sampling pairs. As the final results include variation of the atmosphere in the bioaerosol chamber, differences between the two sampling pumps, and the used analysis method, the influencing factors cannot be considered separately. For the first evaluation, the paired samples (taken in the front and the back) were plotted against each other, and the Spearman correlation coefficient was calculated (see Fig. S3; Table 5).

As this first evaluation showed that the used analysis method had a strong influence on the observed variation, we compared the variances between the different analysis methods. Ideally, the CV is calculated based on a multitude of similar samples. In our setup, the samples were spread over a wide range of concentrations, with two sampling replicates in exactly the same atmosphere. Therefore, the formula was transformed to account for the different atmospheres from which each sampling pair was taken:


$$CV=\frac{\sigma }{\mu }=\frac{\sqrt{\frac{1}{N}\sum {\left(x_{i}-\bar{x}\right)}^{2}}}{\bar{x}}=\sqrt{\frac{1}{N}}\sqrt{\sum {\left(\frac{x_{i}-\bar{x}}{\bar{x}}\right)}^{2}}$$

$$CV_{sampling\;pairs}=\sqrt{\frac{1}{N}}\sqrt{\sum {\left(\frac{x_{i}-{\bar{x}}_{sampling\;pair}}{{\bar{x}}_{sampling\;pair}}\right)}^{2}}$$

This CV was calculated for each analysis method and organism individually, as well as overall organisms for this analysis method.

#### Used programs and functions

To process the data, generate plots, and calculate correlations, the following programs were used: Microsoft Excel 365, LibreOffice calc, and R 4.4.1 (40). In R, three packages with the following functions were used: *base*: plot(), summary(), mean(); *graphics*: boxplot (); *stats*: cor.test(x, method = c("spearman")), t.test(), shapiro.test(), sd(), aggregate(), quantile();

## RESULTS

### Influences of aerosol type on quantification

**Fig 1:**
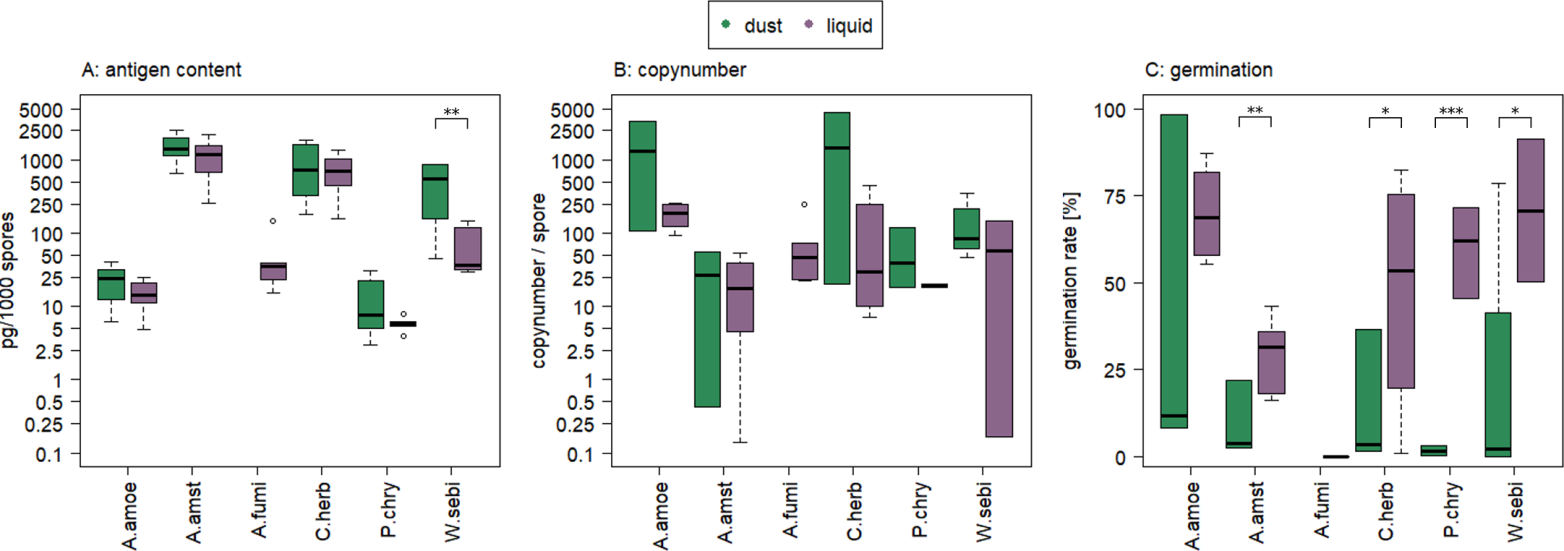
Antigen content per 1,000 spores, germination rate, and copy number per spore of the starting material before aerosol production; green—dry material for the RBG 1,000; violet—liquid suspension for the LSA; due to safety reasons, no dust of *Aspergillus fumigatus* (A. fumi) was produced; Abbreviations: *Aspergillus amoenus* (A.amoe), *Cladosporium herbarum* (C. herb), *Penicillium chrysogenum* (P. chry), *Wallemia sebi* (W. sebi); significant differences (t-test) are indicated by ***P < 0.001, **P < 0.01, *P < 0.05.

To create the 172 aerosol samples from six fungal species, two different methods of aerosol production were used, starting either with an aqueous spore suspension or with dry spore dust. From this source material, aliquots were analyzed using the different detection methods. The results are illustrated in Fig. 1.

For ELISA-based antigen content, no strong influence of the method of spore production on subsequent quantification could be identified (t-test, *P* = 0.249), except for *Wallemia sebi* (t-test, *P* = 0.007, Fig. 1A). Copy numbers/spore (see Formula 2), were in most cases higher in the dry material than in the liquid source material, but the difference was not significant (t-test, *P* > 0.05, Fig. 1B). By contrast, germination rates of dry material were significantly lower than those of the liquid material for four of the fungal organisms (Fig. 1C), most likely due to reduced cultivability from freeze drying.

Among the tested species, larger-spored fungi (*Aspergillus amstelodami* and *Cladosporium herbarum*) exhibited higher antigen content/1,000 spores, while smaller-spored fungi (*Aspergillus amoenus*, *Aspergillus fumigatus,* and *Penicillium chrysogenum)* showed lower values (Fig. 1A). However, *Wallemia sebi* was an exception, displaying high antigen content/spore, especially when measured in dried material, despite having spores similar in size to *P. chrysogenum* or *A. amoenus*.

### Reproducibility of sampling and analytical methods

#### Reproducibility of analytical methods

All methods for quantification of samples, except spore counting, were carried out in technical replicates, and the coefficient of variation (CV) of each set of technical replicates was calculated, and the results are summarized in Table 4.

**TABLE 4:** Variation of analysis results of technical replicates for four detection methods (dilution series in ELISA, three plates for cultivation, and four wells for qPCR)^*a*^

Fungal source material	Mean % CV (10–90 percentile)			
	ELISA antigen using mixing extraction	ELISA antigen using cell disruption	Cultivation CFU	qPCR copy numbers of specific assays
*Aspergillus amoenus*	18.97 (0–30)	20.4 (0–33)	31.6 (7–73)	*Aspergillus* 19.9 (5–41)
*Aspergillus amstelodami*	9.4 (3–16)	10.6 (3–18)	52.4 (9–106)	
*Aspergillus fumigatus*	3.2 (0–16)	4.3 (0–17)	116.3 (87–156)	
*Cladosporium herbarum*	4.0 (0–10)	3.9 (1–9)	39.6 (3–99)	*Cladosporium* 48.4 (12–93)
*Penicillium chrysogenum*	0.1 (0–0.1)	0.1 (0–0.2)	23.1 (6–39)	*Penicillium* 23.1 (9–43)
*Wallemia sebi*	17.3 (5–36)	20.3 (6–39)	62.3 (6–173)	*Basidomycetes* 3.3 (1–9)
All samples	10.0 (0–26)	10.1 (0–29)	36.75 (3–89)	FungiQuant 37.2 (8–81)
Number of analyses	*n* = 405	*n* = 405	*n* = 291	*n* = 363

^*a*^ Numbers of analyses include all kinds of samples (starting material, samples directly after aerosolization, and filters) and are different for each method, as not all samples were above the respective detection limit.

The last row of Table 4 shows the number of samples analyzed for each method. The sample counts differ between methods due to two factors: samples below the detection limit for certain methods and the use of multiple assays for mixed samples (five ELISAs, general and specific primers for qPCR). Specifically, no colony-forming units (CFUs) could be determined from *Aspergillus fumigatus*, because the spore suspension was treated with fungicide prior to aerosolization for safety, rendering the spores non-viable.

Considering all samples as well as results for most single organisms, the results show lower mean CVs for ELISA compared to cultivation and qPCR. Only the Basidiomycetes primer set (see row of *Wallemia sebi*) shows a lower mean CV than the respective ELISA. For fungal detection with ELISA, two extraction methods for the samples were used: mixing and cell disruption. The respective columns show that cell disruption resulted in slightly higher mean CVs than mixing extraction, with no statistically significant impact. Results from cultivation and qPCR range up to a maximum of 200% variation.

Due to time constraints on the experimental day and the need to perform spore counting without delay after filter extraction, spore concentration was analyzed only once for each sample. The personal counting error of the counting technician was evaluated by counting 10 aliquots of a *Penicillium chrysogenum* spore suspension (7 × 10^6^ spores/mL), resulting in a relative CV of 7.4%.

#### Reproducibility of sampling

For each of the created environments with different spore concentrations, two air samples were collected simultaneously, except for *Aspergillus amoenus and Aspergillus fumigatus*, for which another sampling device was compared to the filtration system. In some rare cases, three samples were taken simultaneously, leading to uneven sample numbers (See number of samples in the last row of Table 5).

**TABLE 5:** Coefficient of variance (in %) for four detection methods and four fungal organisms, based on sampling pairs as described in the formula CV sampling pairs^*a*^

	Fungal source material	Spore counting	ELISA antigen using mixing extraction	ELISA antigen using cell disruption	Cultivation CFU	qPCR copy numbers (FQ primer)
CV of simultaneous samples (%)	*Aspergillus amstelodami*	17.23	13.30	12.54	41.21	67.67
	*Cladosporium herbarum*	18.44	18.50	18.28	45.34	65.20
	*Penicillium chrysogenum*	13.67	16.67	11.23	33.30	65.96
	*Wallemia sebi*	19.26	20.66	21.70	57.40	73.11
CV over all samples (%)		17.79	15.25	14.75	39.22	59.19
Spearman over all samples		ρ = 0.989	ρ = 0.986	ρ = 0.983	ρ = 0.882	ρ = 0.578
Number of samples		94	117	111	99	111

^*a*^ Only groups with single organisms and simultaneous sampling are included. Numbers of included samples for each method deviate, as not all samples were above the detection limit for all methods.

Based on the results of simultaneously taken samples, a coefficient of variation across all concentrations was calculated for each different detection method. These CVs include the variability of the sampling and the analysis itself. In addition, the correlation coefficient after Spearman was calculated (Table 5; Fig. S3).

The results from simultaneous samples show a good correlation and a low CV for ELISA and spore counting. For the detection method of cultivation, the deviation of the analyses was higher, and therefore the correlation was lower (see respective columns in Table 5). In this study, the deviation with qPCR was the highest, most probably resulting from variability introduced during DNA extraction in 96-well plates.

Comparing the two extraction methods used before ELISA measurement, the CVs were similar, and there was only a minor difference in correlation (see respective columns in Table 5). Also, the variance of sampling pairs analyzed by spore counting was similar to the variance from the various ELISAs. The same was true for the correlation coefficient (see row Spearman of Table 5; Fig. S3).

### Comparison of extraction methods for ELISA

**Fig 2:**
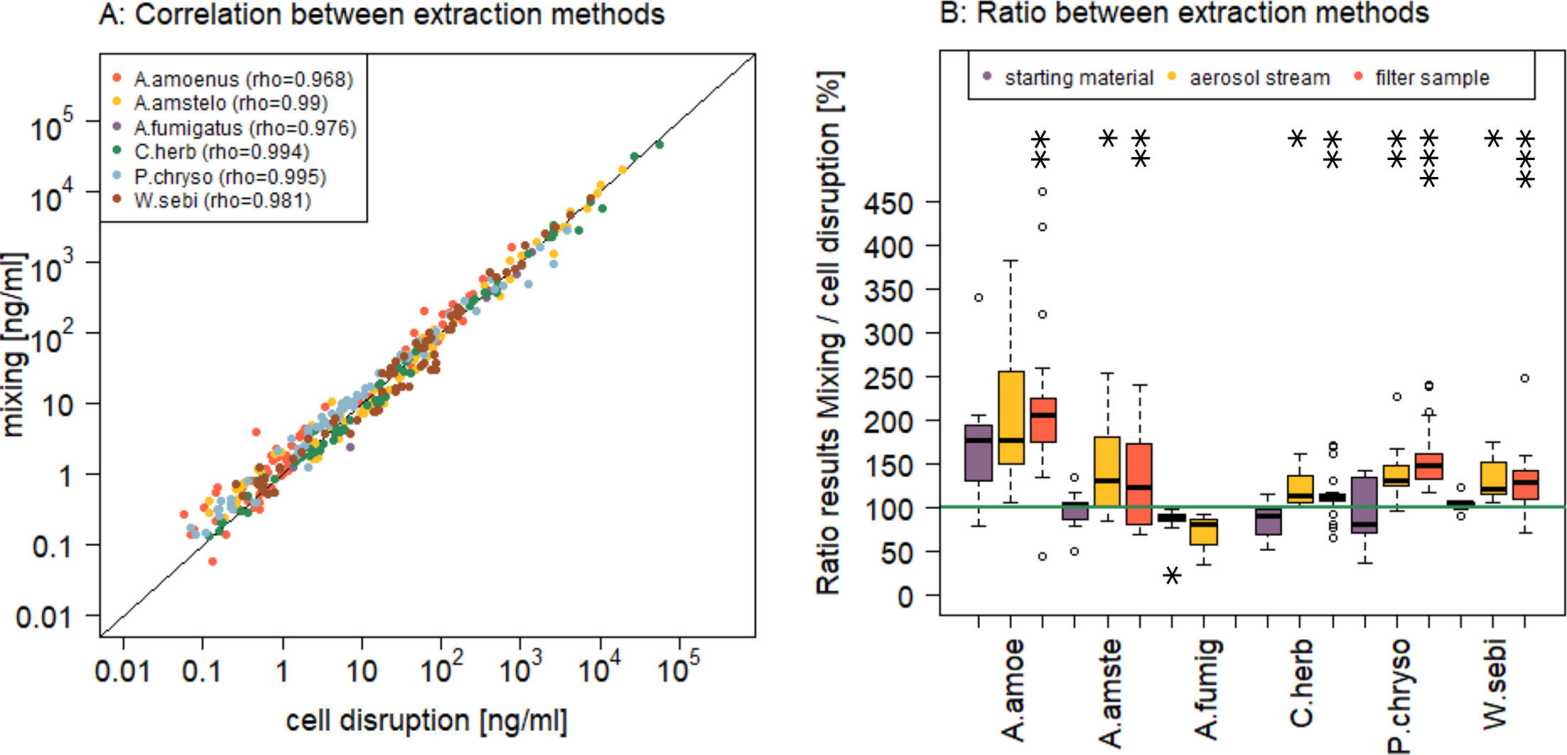
ELISA results of all suspensions, comparing liquid extraction and cell disruption, *n* = 384. (A) Correlation of the extraction methods, indicating the different organisms by color; (B) portion of the results of cell disruption to mixing extraction, green line indicating the same result; violet—starting material, yellow—collected aerosol stream, red—solution of filter extraction after aerosol sampling; significant differences to 100 (t-test) are indicated by placement below or above the line as *** *P* < 0.001, **P < 0.01, *P < 0.05.

To extract antigens from each sample across each stage of aerosol production (starting material, samples directly after aerosolization, and filter samples), two extraction methods were used and the results compared. One extraction was done by mixing the sample using a vortex, whereas the other extraction included a bead-beating step, disrupting the cells. The results show a high correlation (ρ ≥ 0.97) between the two extraction methods (Fig. 2A). A closer look reveals that depending on the organism, one of the extraction methods results in higher ELISA results, with the most profound difference for *Aspergillus amoenus*, where mixing extraction gave results about double of the cell disruption results (Fig. 2B). The differences in favor of mixing were more pronounced in the filter samples (paired t-test, *P* < 0.001) than in the starting material (paired t-test, *P* > 0.05).

### Minimal spore numbers for detection in ELISA

Besides the optimal antigen extraction method, we also investigated the limit of detection of the fungal-specific ELISAs based on spore concentration. The limit of detection based on protein concentration had been obtained in previous studies (Table 3). To evaluate how many spores correlate with this protein limit of detection, we used the measured antigen concentrations and counted spore numbers from samples, as well as the protein quantification limit for calculation. The results are presented in Table 6. Individual biological samples and values close to the detection limits of the methods showed large deviations. To exclude outliers, the 10%–90% percentile is therefore given instead of the range.

**TABLE 6:** Minimum spore number for detection in specific ELISAs based on detection limits presented in Table 3, calculated for all extracts with a spore concentration above the quantification limit of spore counting

Fungal source material	Extraction by mixing only		Extraction by cell disruption	
	Sample number [*n*]	Median (10–90 percentile) [spores/mL]	Sample number [*n*]	Median (10–90 percentile) [spores/mL]
*Aspergillus amoenus*	42	7,966 (3,114–27,814)	40	16,462 (5,452–56,772)
*Aspergillus amstelodami*	33	20 (7–254)	33	19 (8–606)
*Aspergillus fumigatus*	12	6,083 (3,046–12,438)	11	4,870 (2,796–11,510)
*Cladosporium herbarum*	31	31 (14–133)	31	35 (15–135)
*Penicillium chrysogenum*	46	11,855 (5,429–17,562)	43	16,362 (10,086–20,909)
*Wallemia sebi*	41	3,173 (408–5,740)	41	3,985 (599–6,969)

The data show differences between fungal species, ranging from a median of 20 to nearly 16,500 spores/mL required to obtain a valid ELISA signal (see Table 6). The species with larger spores, *A. amstelodami* and *C. herbarum*, needed fewer spores/mL for the detection than species with smaller spores. Although *W. sebi* also has small spores, lower spore numbers were needed than for *A. amoenus* or *A. fumigatus*. Moreover, the results show that the calculated detection limit of the ELISAs for *A. amstelodami* and *C. herbarum* was below the detection limit of spore counting (ca. 5,000 spores/mL). In addition, results show variant results for the material of the same fungal strain.

### Results of single fungal air samples with the different detection methods

For each test organism, five different settings were used for aerosol production. Filter extracts were analyzed with four different detection methods (see Table 7), and correlations between the detection methods were calculated (see Table 8).

**TABLE 7:** Overview of results from four different methods for measuring filter samples prepared by aerosolizing six different fungi with different settings, with intended increase in spore concentration^*a*^

Setting	Fungal source material	Spore counting spores/µL		Cultivation CFU/µL		qPCR copy number/µL		ELISA antigen [pg / µL] mixing extraction		ELISA antigen [pg/µL] cell disruption		Germination rate spores/CFU [%]	
		Median	Range	Median	Range	Median	Range	Median	Range	Median	Range	Median	Range
1	*A. amoenus*	5.00	1.11–7.77	1.33	0.43–4.10	1,795.34	9.82E + 02–1.73E + 05	0.10	0.06–0.22	0.13	0.07–0.15	32.50	14.75–121.51
2	*A. amoenus*	5.56	1.11–15.56	1.75	0.01–4.73	3,234.77	7.35E + 02–4.15E + 03	0.25	0.11–0.65	0.09	0.06–0.15	32.55	0.10–129.01
3	*A. amoenus*	373.00	102.00–6500.00	51.33	19.67–132.33	134,035.86	3.74E + 04–1.36E + 06	2.32	0.80–101.26	1.15	0.43–46.28	19.28	16.54–29.60
4	*A. amoenus*	470.00	42.10–1040.00	139.00	24.33–351.33	237,408.33	1.05E + 05–3.99E + 05	3.97	1.71–8.77	1.11	0.47–3.39	29.57	16.78–57.80
5	*A. amoenus*	480.00	200.00–850.00	53.33	10.67–75.33	183,632.77	1.18E + 05–4.33E + 05	3.04	1.57–4.05	1.52	1.10–1.91	13.49	1.25–27.10
1	*A. amstelo*	0.00	0.00–0.00	0.02	0.00–0.03	32.79	3.28E + 01–3.28E + 01	0.36	0.35–0.36	0.25	0.24–0.26	0.00	0.00–0.00
2	*A. amstelo*	14.05	1.10–50.00	0.25	0.07–0.37	11,966.05	2.40E + 01–8.80E + 04	1.12	0.732.71	0.52	0.34–2.24	17.25	0.37–33.33
3	*A. amstelo*	1.65	1.10–34.00	0.20	0.04–1.13	12,666.13	3.45E + 01–5.25E + 04	3.40	0.28–6.91	2.44	0.12–6.30	18.18	0.10–66.67
4	*A. amstelo*	98.00	40.00–197.00	2.33	0.33–38.67	17,418.75	4.64E + 02–2.42E + 06	61.06	45.56–114.43	65.87	48.25–82.71	2.38	0.44–19.83
5	*A. amstelo*	22.00	12.00–253.00	1.27	0.73–6.00	17,378.77	2.34E + 02–6.53E + 05	7.40	7.13–60.60	10.43	9.64–77.10	3.21	2.16–10.95
1	*A. fumigatus*	47.78	27.78–64.00	0.00	0.00–0.00	253.10	1.00E + 02–3.99E + 02	0.00	0.00–0.00	0.00	0.00–0.00	0.00	0.00–0.00
2	*A. fumigatus*	46.67	20.00–67.78	0.00	0.00–0.00	286.38	9.45E + 01–2.13E + 03	0.00	0.00–0.00	0.00	0.00–0.00	0.00	0.00–0.00
3	*A. fumigatus*	95.00	75.56–107.00	0.00	0.00–0.00	652.90	3.66E + 02–1.71E + 03	1.20	1.20–1.20	2.08	2.08–2.08	0.00	0.00–0.00
1	*C. herb*	1.11	1.11–1.11	0.10	0.00–0.33	403.31	1.25E + 02–1.71E + 07	0.87	0.49–3.98	0.80	0.37–2.32	10.07	9.00–9.00
2	*C. herb*	3.89	2.22–5.50	1.43	0.17–2.83	21,308.54	7.43E + 02–9.56E + 05	1.58	0.60–1.80	1.49	0.56–1.71	14.64	7.50–85.01
3	*C. herb*	287.00	43.00–340.00	6.37	0.63–65.00	74,861.55	0.00E + 00–2.66E + 06	249.38	18.66–298.76	223.80	16.55–258.50	13.21	0.21–20.31
4	*C. herb*	154.00	48.00–1100.00	15.10	3.67–102.00	9,455.52	0.00E + 00–1.04E + 05	42.43	27.23–363.30	37.20	24.77–377.79	19.73	1.25–32.12
5	*C. herb*	331.00	91.00–386.00	11.67	4.67–31.67	1,612,391.04	3.61E + 05–2.05E + 06	26.59	5.66–27.79	34.50	7.34–41.30	5.13	3.02–9.57
1	*P. chryso*	10.00	1.00–23.00	5.40	0.70–16.83	752.20	2.26E + 02–1.16E + 06	0.24	0.15–0.33	0.16	0.11–0.16	54.00	51.45–76.67
2	*P. chryso*	20.00	7.00–28.00	15.33	1.77–25.33	471.08	9.05E + 01–1.16E + 08	0.39	0.16–0.45	0.17	0.07–0.22	23.48	23.48–110.14
3	*P. chryso*	1,200.00	370.00–2700.00	51.10	11.53–151.00	46,262.62	9.81E + 03–1.16E + 07	6.59	1.80–13.52	5.12	1.47–10.51	2.49	1.05–12.48
4	*P. chryso*	2,175.00	1620.00–5500.00	129.67	32.00–696.00	183,504.48	4.36E + 03–1.16E + 08	14.71	10.50–50.19	10.00	7.00–31.15	6.96	0.93–36.63
5	*P. chryso*	8,000.00	7000.00–9600.00	51.00	48.00–54.00	227,130.21	1.68E + 05–3.00E + 05	46.92	43.95–48.24	32.90	30.23–39.08	0.57	0.50–0.64
1	*W. sebi*	5.78	3.33–7.78	2.86	2.71–3.27	4,248.77	1.89E + 03–2.02E + 04	0.72	0.51–1.28	0.56	0.32–0.80	56.51	36.29–98.01
2	*W. sebi*	18.89	12.00–40.00	6.93	3.10–11.50	21,945.05	2.57E + 03–1.22E + 09	0.76	0.53–1.25	0.61	0.50–0.94	33.94	19.93–55.56
3	*W. sebi*	453.50	408.00–506.00	0.33	0.00–2.00	2,416.63	1.52E + 03–7.52E + 04	26.94	25.23–31.00	22.33	18.44–25.86	0.07	0.00–0.48
4	*W. sebi*	1,310.00	81.10–1430.00	3.33	0.00–6.67	79,042.77	1.41E + 03–2.42E + 06	80.51	39.72–170.71	68.17	27.06–130.90	0.23	0.00–0.50
5	*W. sebi*	2,315.50	943.00–2600.00	3.33	0.00–221.25	207,021.29	1.67E + 03–1.22E + 09	151.56	62.77–229.84	146.07	47.06–177.73	0.16	0.00–9.22

^*a*^ For each setting and fungal species, at least five filters were prepared, processed, and analyzed; settings 1 and 2 (and three in case of *A. fumigatus*) used liquid material, 3–5 used dry material for aerosolisation, total number of filter samples analyzed = 172.

**TABLE 8:** Correlation coefficient after Spearman (ρ) for the comparison of the detection methods to each other, given for the samples resulting from dry and liquid starting material^*a*^

	Dry starting material				Liquid starting material			
	Cultivation	qPCR	ELISA mix	ELISA cd	Cultivation	qPCR	ELISA mix	ELISA cd
*A. amoenus*								
Spore counting	0.63	**0.90**	0.84	0.81	0.87	0.81	0.68	0.88
Cultivation		0.52	0.63	0.50		0.86	0.62	**0.92**
qPCR			0.80	0.85			0.63	**0.93**
*A. amstelodami*								
Spore counting	0.81	0.25	**0.91**	**0.91**	0.79	0.15	0.75	0.73
Cultivation		0.11	0.66	0.74		0.55	**0.93**	**0.92**
qPCR			0.17	0.23			0.52	0.54
*A. fumigatus*								
Spore counting						0.87	**0.91**	0.86
Cultivation								
qPCR							**0.97**	0.80
*C. herbarum*								
Spore counting	0.64	0.67	0.85	**0.90**	0.87	0.65	0.70	0.75
Cultivation		0.45	0.53	0.57		0.34	0.82	0.86
qPCR			0.51	0.57			0.17	0.32
*P. chrysogenum*								
Spore counting	0.26	0.81	**0.97**	**0.98**	**0.95**	0.38	0.74	0.80
Cultivation		0.21	0.30	0.73		0.35	0.83	0.87
qPCR			0.84	0.85			0.49	0.77
*W. sebi*								
Spore counting	0.27	0.49	0.65	0.66	**0.93**	0.34	0.77	0.77
Cultivation		0.02	0.06	0.06		0.14	0.85	0.79
qPCR			0.63	0.61			0.25	0.26

^*a*^ Gray, ρ ≥ 0.75; bold, ρ ≥ 0.9; cd, cell disruption; mix, mixing extraction.

#### Quantification results of different methods

The five aerosolization settings used aimed to create ascending concentrations in the air and the subsequent filter samples (see “Aerosol production,” above). This was successful for *A. amoenus*, *P. chrysogenum,* and *W. sebi*. For *A. amstelodami,* the results show a drop in the median of setting 5, giving results between settings 3 and 4 (see Table 7, spores/µL). Furthermore, this fungus showed inconsistent spore output for liquid aerosolization, resulting in high deviations in the respective concentrations and no stable gain of aerosol concentration (compare *A. amstelodami* settings 1–3 in Table 7, spore counting and cultivation). For *C. herbarum*, the fourth setting resulted in numbers below the third and fifth setting (see results for spore counting and qPCR). For *A. fumigatus*, only three settings were possible, as no dry dust could be produced for safety reasons (see “Aerosol production,” above). Spore concentrations for settings 1 and 2 were close together (see Table 7).

The results for spore counting show that the range of spore concentration differed for each of the fungal species used as source material. Higher concentrations of spores on the filters were necessary for some of the organisms to reach the detection limit of the ELISAs (see Table 7, spore counting and ELISA). Setting one was intended to be at the detection limit. For *Aspergillus amstelodami,* this meant that it was below the concentration of spores that could be counted. Therefore, no spore count is given for this concentration. For *Aspergillus fumigatus,* it was not possible to produce ascending concentrations in the detection range of the specific ELISA. Only with the highest concentration producible with the liquid spore material, enough spores could be brought into the aerosol chamber to result in sufficient amounts of antigen in the extracts.

For nearly all fungal species, the germination rate of samples was derived from fresh (liquid) material. Germination rates for settings one and two were clearly higher when compared to the germination rates from dried spores (dust), used in settings three to five (see Table 7, germination rate).

#### Correlation between detection methods

As we analyzed each sample with the four different detection methods, results for each sample aliquot could be directly compared, and Spearman correlation coefficients were calculated to evaluate agreement between methods (see Table 8). As the cultivability was reduced through the production of spore dust, the correlation coefficient was calculated for samples derived from fresh material (liquid suspensions) and dry material (spore dust) separately, see columns of Table 8.

Considering all the results given in Table 8, the various ELISAs show a good correlation to spore counting. For the liquid starting material, the correlation to cultivation was mostly better than to spore counting. For *Wallemia sebi*, the data show a clear difference between the dry and liquid starting material in the correlation of cultivation and ELISA results.

### Testing of mixed samples

**Fig 3:**
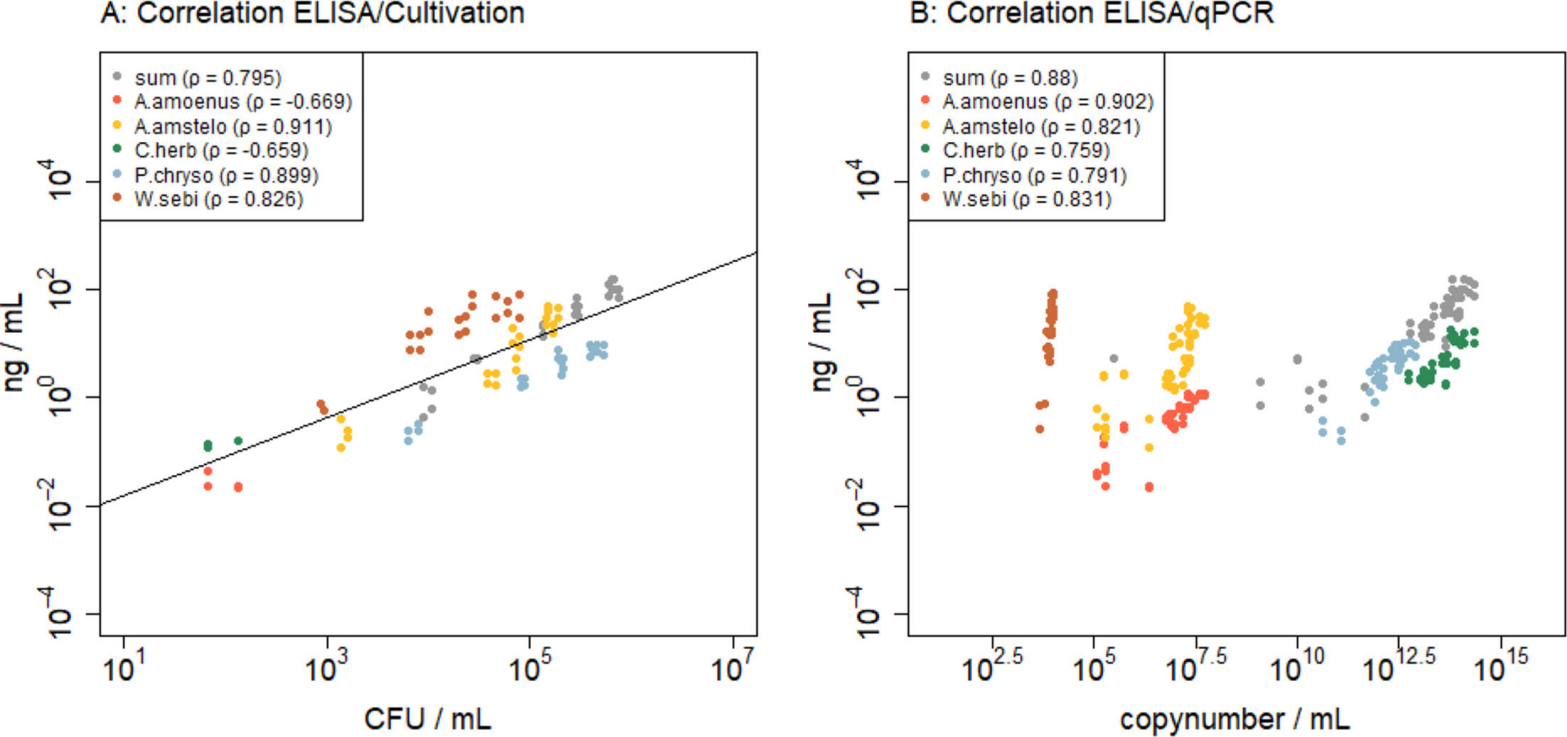
Comparison of ELISA results with results of other detection methods for filter samples derived from mixed fungal source material; (A) Correlation between methods ELISA and Cultivation; (B) Correlation between methods ELISA and qPCR; *Aspergillus amstelodami* (A. amstelo), *Cladosporium herbarum* (C. herb), and *Penicillium chrysogenum* (P. chryso).

Since aerosols of individual fungal species do not occur in the natural environment, we generated an aerosol mix of five fungal species in the bioaerosol chamber (Fig. 3). In preparation for these tests, we evaluated whether the fungal-specific ELISAs react with non-target fungal species using non-aerosolized liquid spore suspensions (Table 9).

**TABLE 9:** Relative reactivity of spore extracts in species-specific ELISAs^*c*^

Fungal source material	Reactivity %											
	*Aspergillus amoenus* ELISA		*Aspergillus amstelodami* ELISA		*Aspergillus fumigatus* ELISA		*Cladosporium* ELISA		*Penicillium chrysogenum* ELISA		*Wallemia sebi* ELISA	
	Prot^*a*^	Spore^*b*^	Prot	Spore	Prot	Spore	Prot	Spore	Prot	Spore	Prot	Spor
*Aspergillus protuberus*	100	100	0.44	0.06	<0.09	<0.02	0.01	0.003	25	2.8	**293**	36
*Aspergillus amstelodami*	4.3	20.6	100	100	<0.19	<0.19	0.04	0.04	**152**	**166**	39	23
*Aspergillus fumigatus*	<0.37	<0.90	0.09	0.09	100	100	0.02	0.01	4.9	5.5	0.52	0.31
*Cladosporium herbarum*	0.16	0.75	0.14	0.07	<0.13	<0.25	100	100	<0.07	<0.14	0.31	0.36
*Penicillium chrysogenum*	0.72	1.58	0.12	0.11	<0.06	<0.06	0.005	0.004	100	100	2.62	1.39
*Wallemia sebi*	0.42	3.46	0.004	0.01	0.22	0.38	<0.003	<0.002	0.14	0.26	100	100

^*a*^ Based on the same protein concentration as the target organism.

^*b*^ Based on the same spore concentration as the target organism.

^*c*^ Gray, reactivity higher than 20%; bold, reactivity higher than 100%. Homogenization was performed by bead beating, except for *Wallemia sebi*, which was prepared by mixing extraction, matching the establishment of the ELISA (see references in Table 3).

#### Cross-reactivity of ELISA tested in liquid spore suspensions

To evaluate cross-reactivity between ELISAs and non-target fungal species, the results are presented relative to the reactivity of the target-specific ELISA (Table 9). In these experiments, *A. protuberus* was used as a representative of the clade *Aspergillus versicolor*, since at the beginning of our study, the standard of the *Aspergillus versicolor* ELISA had not yet been sequenced and identified by us as *Aspergillus amoenus*.

To compare the reactivities, the values were normalized either to equal protein concentrations or to equal spore concentrations (Table 9).

The results show that there was little to no cross-reactivity for most of the ELISAs with most of the spore extracts (Table 9). However, we found high cross-reactivity between the *Penicillium chrysogenum* ELISA and *Aspergillus amstelodami* extract as well as between the *Wallemia sebi* ELISA and *Aspergillus protuberus* extract*,* which exceeded the reactivity of the target spore extracts (Table 9).

#### Testing mixed aerosols with all analysis methods

To conclude the experiments and compare the four different detection methods, mixed fungal material as spore suspensions and dust was produced from BSL-1 organisms. The material was aerosolized in different settings, sampled, processed, and analyzed. In comparison to the single-organism samples, spore counts were not available for single organisms, as only *Aspergillus amstelodami* and *Cladosporium herbarum* spores can be clearly distinguished in this spore mixture under the microscope. The other three organisms have optically similar spores and could not be identified quantitatively. Therefore, results from spore counting were compared to cultivation (CFU), ELISA (antigen), and qPCR (copy number) based on the sum of all fungal species.

Results show a high correlation of spore counts to cultivation and ELISA, with ρ = 0.93 and 0.97, respectively. The method used for sample preparation (bead beating versus liquid extraction) made no difference in the correlation coefficient, with 0.973 and 0.975, respectively. Correlation of spore counting to qPCR was lower but still in a high range (ρ = 0.76).

For the methods where distinction between species was possible, scatter plots were created, and the Spearman correlation coefficients were given (Fig. 3). When comparing cultivation to ELISA (Fig. 3A), most data correlate well (ρ > 0.8) and are arranged along the theoretical line of perfect correlation. For *A. amoenus* and *C. herbarum,* as only a few data points are available, only a few CFU were countable, resulting in a negative correlation coefficient (Fig. 3A).

Comparing qPCR and ELISA data (Fig. 3B), the correlation coefficients of all fungal species were high (ρ > 0.76), with only *C. herbarum* and *P. chryogenum* below ρ = 0.8. Overall, the points are distributed over a broad range of magnitudes, ranging from about 10^3^ to 10^15^ copy numbers/mL, where points of single organisms are mostly within two magnitudes of concentration. In addition, the dot clouds of the same species cluster together, depicting a line with a positive slope. As the gains of these slopes are highly different, they were not included in Fig. 3 but depicted for each single species in Fig. S4. Evaluation of this linear regression shows that it is highly significant (*P* ≤ 0.001) for all species; however, the slopes are partly very steep, and the dots diverge from a perfect correlation line, with an R² between 0.42 and 0.74 (see Fig. S4).

## DISCUSSION

Although there are currently many different methodologies to analyze and quantify airborne fungal spores, there is still a lack of a dose-response relationship to close the gap between exposure and health outcomes (41, 42). Real-time methodologies aim for continuous measurement and the generation of more comprehensive data sets, including differentiating the shape and size of spore agglomerates, influencing the particle penetration into the respiratory tract. However, these methods are limited to easily identifiable targets and may not be able to distinguish between closely related species or morphologically similar spores (30, 43). In addition, research linking exposure and health outcomes and suggesting adequate dose-response relationships is still missing (7). Reasons for the complex connection between concentration and health outcomes caused by fungi also lie in various pathomechanisms for health effects and different susceptibility of individuals (44). Some fungi are infectious and can cause mycoses, while others are responsible for different types of allergies or mucosal irritation. For *Aspergillus fumigatus*, combinations of these adverse health effects have been documented (44, 45).

As the detection of airborne propagules instead of the quantification of immunologically reactive substances, like mycotoxins, does not provide a complete picture of exposure, immunological methods have been developed to measure antigens and/or allergens. However, various analytical methods have been published and used in studies to assess the concentration of allergen-producing fungi. In the study presented here, we evaluated the applicability of six fungal-specific ELISAs based on polyclonal antibodies and compared them with different quantification methods used in aerosol studies.

### Considerations about the test setup

The test system used has been used already in other studies testing analytical methods or bioaerosol sampling devices (11, 17, 25); however, in the mentioned studies, only one or two concentrations of each specific aerosol were produced. In the study presented here, multiple concentrations were produced for each fungal aerosol, and only two sampling systems of the same type were used in the same atmospheric conditions. Therefore, we used a modified formula for the calculation of the coefficient of variance, using the mean of the sampling pairs as the basis (see “Statistics and calculations,” above) for each analytical method. The results show a high influence of the detection method on the obtained CV, with high numbers for CFU determination (about 40%) and molecular genetic analysis (about 60%), in contrast to lower results for spore counting and ELISA, ranging between 13% and 22%. In comparison, in another study, the analysis of multiple samples of aerosolized endotoxin-containing dust with subsequent analysis of endotoxin activity via the rFC method revealed an even lower CV between 8% and 12% (25). The reason for these discrepancies may lie in the difference in sample processing and analytical methods, as strong variations between the analytical methods used here could also be observed.

In the experiments presented here, two types of aerosol generators were used, including different methods for material production (liquid versus dry). As a first step, we evaluated how the different source materials were recognized by the analytical methods without producing aerosols. The results show a strong reduction in germination rate when producing dry spore dust, most likely due to the stress acting on the spores in the environmental changes during freezing (46) but also drying. The results show that the antigen content in 1,000 spores, as measured by ELISA, was not influenced by the different preparation of the source material, indicating that the antigen content was not significantly impacted by freeze-drying and storage of the material at room temperature, and the antigen content does not depend on the ability of spores to germinate (viability). The only exception is the used Basidomycota, *Wallemia sebi*. The reason for the change in antigen content after freeze-drying is not known and would need further investigation. These results are especially important, as even spores not detectable by cultivation have antigens and may lead to allergic reactions. However, as the ELISAs used in this study are based on polyclonal antibodies and directed to a mixture of antigens of the respective organism as immunogen, no specific allergen is targeted; therefore, the impact of this discrepancy for clinical considerations is not known. Further evaluation is hindered as no single allergen of *Wallemia sebi* has been identified so far.

Nevertheless, the discrepancy between the recognition of fresh and freeze-dried spores by the fungal-specific ELISAs may lead to discrepancies in the quantification of airborne *Wallemia sebi* in field studies, depending on the growing conditions or dry state of the culture. The same applies to other fungi when only cultivation is used for detection in the field, as dust with non-germinating fungal particles will not be detected, even though it contains antigens.

The comparison of the germination rate and antigen content would be especially interesting for *Aspergillus fumigatus*, as this species can grow at 37°C and therefore may lead to infections in immunocompromised individuals, as well as it can lead to allergic reactions. However, this study was conducted in a bioaerosol facility with biological safety level one, which did not allow us to work with living and potentially infectious *Aspergillus fumigatus* spores.

### Extraction protocol assessment

Producing multiple aliquots of the same filter extracts enabled the testing of two different sample processing protocols prior to fungal-specific ELISAs. The comparison of mixing extraction and cell disruption showed a high correlation for all tested species. The quantitative data showed only for *A. fumigatus,* a clear higher output when cell disruption was included, and for *A. amoenus,* higher concentrations for liquid extraction; however, the differences were at most less than a factor of 2.

The comparison of the methods suggests that it is not necessary to use cell disruption in routine analysis applications. As cell disruption requires more material and time for sample processing, this may reduce costs associated with the ELISA analysis. However, if the filter extract is processed for different analytical methods, for example, using bead beating for DNA extraction, our results suggest that it is not necessary to prepare separate aliquots for ELISA analysis without cell disruption. The same extract can be used for both methods.

On the other hand, it should be considered that the aim of using antigen-recognizing methods is to move from particle measurement closer to the measurement of health-related agents. As human epithelial cells do not conduct cell disruption, processing of samples by mixing extraction may better reflect the exposure relevant to the immune system.

### Comparison of analytical methods

As many different methods are used for bioaerosol collection and analysis in various environments, research fields, and study designs, a comparison of these methods is necessary. In our results, we show that the fungal-specific ELISAs gave results correlating with obtained spore counts and colony-forming units for single-organism aerosols as well as for mixed samples. However, the power of the correlation varied between the fungal test species and was influenced by the preparation of the aerosol material (liquid vs. dry preparation). This may be due to the low concentration of spores in the liquid suspension, and therefore the spore count being on its own detection limit. The difference in correlation between ELISA and cultivation, for liquid and dry starting material, is especially clear for *Wallemia sebi*. This is most probably due to the very low CFU count of the dry spore dust and the low calculated germination rate (<1%) for this material.

Considering the limit of detection further, and comparing ELISA results to microscopic spore counts, the data revealed that for low concentrations of spores in the filter extracts, the detection limit for ELISA analysis was lower than the detection limit for cell counting. Especially for the fungal species with bigger spores, *Aspergillus amstelodami* and *Cladosporium herbarum*, the respective specific ELISA showed a detection limit below 100 spores/mL, making the assays valuable and sensitive analytical methods.

As the calculated limit of detection was based on the spore counts, these numbers were influenced by the counting error of the counting chamber, the technician performing the analysis, dilution errors, as well as possible differences in reactive protein concentration, as the suspensions were biological replicates. Therefore, only a range for the limit of detection based on spore counts could be given in this study.

When comparing the results obtained by fungal-specific ELISAs to copy number evaluation using qPCR, the data show very different proportions and correlations for the different fungi, with the highest agreement for the *Aspergillus* species. Note that different fungal species possess different numbers of rDNA copies in their genome, which vary between 11 and 251 copies (47). As closely related species are estimated to have similar copy numbers, it can be assumed that the test organism *Aspergillus amoenus* has about 70 rDNA copies per genome. Another factor influencing the comparability of ELISA-based protein detection and copy number quantification using qPCR is the number of cells and their composition forming the fungal spores. While a higher cell number influences both the genome and copy number as well as the antigen content in the same direction, the presence of specific antigens can be very different from the SSU rRNA relevant for qPCR.

Apart from the results for *Aspergillus* test organisms, correlation of ELISA results to the qPCR data, as well as correlation of qPCR data to results from the other analytical methods, is limited. As the standard curves in the qPCR did not show any deviations, we suspect that the sample processing led to variations in the DNA recovery.

Previous studies showed that the bead beating matrix and method, as well as the used DNA extraction kit, may influence copy number enumeration (17). Since the same DNA kit and batch were used for the extraction of all samples, batch differences cannot be responsible for the high deviations of the technical replicates or duplicate determinations. We therefore suspect that the bead beating in a 96-well format was not evenly efficient over the whole plate and introduced variations in DNA yield over the plate. In addition, pipetting of a 96-well plate by hand may also have led to pipetting errors. Based on the results presented here and the data obtained by (17), we emphasize that DNA extraction protocols are designed and optimized for specific samples, easy handling, and high DNA quality, but not necessarily for quantitative DNA extraction. Subsequent to the here-presented study, the protocol used in the laboratory for processing air samples was changed so that samples are processed individually, including freeze-drying, resuspending, bead beating, and centrifugation, before switching into the 96-well plate format and robotic pipetting system.

Considering the reproducibility of the detection methods, the results obtained by qPCR showed a coefficient of variance between 3% and about 50%, depending on the used assay, highlighting again the need for commonly agreed DNA-based methods to ensure higher reproducibility in this field (15). In comparison, most of the fungal-specific ELISAs showed low variations between the technical replicates (1%–11%), whereas cultivation showed the highest variations. As the results derive from the same filter extracts, the higher variation for cultivation and qPCR derives from variation in the detection methods and is not influenced by the bioaerosol chamber and sampling setup. These results emphasize the presented ELISA methods as robust and applicable systems.

### Non-target recognition and mixed samples

The evaluation of the fungal-specific ELISAs by testing them with extracts from the other fungal species revealed cross-reactions which exceeded the reactivity of the target spore extracts between the *Penicillium chrysogenum* assay and *Aspergillus amstelodami* extract, as well as between the *Wallemia sebi* assay and *Aspergillus protuberus* extract*,* although the species are genetically different and not directly related, being of another genus or even another phylum.

As the ELISAs tested here are based on polyclonal antibodies directed to antigen mixtures, they can detect various epitopes on several antigens. Although this makes the tests more sensitive and robust, it also enables binding to multiple epitopes, which increases the likelihood of binding to cross-reacting antigens in other species.

Strategies to overcome cross-reactions in ELISA design include the use of monoclonal antibodies, which limit the ELISA to single epitopes, making it more specific but less sensitive, and/or the use of recombinantly produced specific antigens for antibody production. Nevertheless, even with these strategies, cross-reactions are possible if antigens from other species have similar epitopes.

Moreover, the comparison of species recognition in mixed samples using fungal-specific ELISAs and fungal-specific qPCR assays showed high rank correlation and agreement of the result distributions (see Fig. S4; Table S1), making a point to the usability of the fungal-specific ELISAs for quantification of specific species. Furthermore, the tests emphasize the problems regarding spore counting (optical differentiation) and colony-forming unit evaluation (different growth rates and colony size, introducing overgrowth of small, slow-growing species) in mixed samples, leading to poor correlation of cultivation with the other detection methods for *Aspergillus amoenus* and *Cladosporium herbarum*.

Regarding qPCR and ELISA, the results for the mixed samples show different relations between copy number and antigen content depending on the analyzed species, as has already been seen for single-species aerosols, although other qPCR assays were used (generic for single-species aerosols and specific for mixed aerosols). The results for the concentrations of antigen content and copy numbers are influenced by different factors, varying for each organism and analytical methods. These factors are spore size, protein content/spore, cells/spore, copy numbers per cell or genome, and sensitivity of the different detection methods. However, it must be taken into account that for the two *Aspergillus* species, the same qPCR assay was used. The resulting Ct value was then converted to copy numbers using the standard series specific for either *Aspergillus* species.

### Conclusion

Summarizing our results for testing and comparing fungal-specific ELISAs in a controlled environment, it is possible to draw conclusions regarding their application for routine analysis. Two of the tested fungal-specific ELISAs, for *Aspergillus amstelodami* and *Cladosporium herbarum*, showed very low detection limits regarding the required spore concentration in the sample. Both assays also showed no cross-reactivity with the other tested fungal materials. These ELISAs are therefore well suited for sensitive and specific detection of the respective antigens in environmental samples. On the other hand, the assays for the detection of *Penicillium chrysogenum* and *Wallemia sebi* showed cross-reactions with *Aspergillus* species, making their use in field analysis difficult.

The comprehensive comparison of the sample preparation using cell disruption and mixing extraction showed that the material and time-intensive procedure prior to ELISA measurement is generally not necessary, suggesting the use of mixing extraction for antigen extraction only. The results presented here show a high reproducibility of the ELISA results, demonstrating their applicability in routine analysis. ELISA measurements are comparatively simple and quick compared to cultivation and CFU counts.

Evaluating the other tested methods, spore counting is a reliable way of evaluating the concentration of spores in a liquid suspension; however, the detection limit is relatively high compared to ELISA and CFU analysis. In addition, the tests using mixed fungal material showed that it is not applicable to distinguish similar-looking spores in a suspension, even when only five species are mixed and the typical spore sizes and forms are known. Although statistically, *Wallemia sebi* has smaller spores than *Aspergillus amoenus*, it was not possible to distinguish these species quantitatively in the mixed suspension due to blurred size limits of both species with round spores. This illustrates that spore counting in environmental samples is limited to easily recognizable and predominantly large spore-forming species such as *Alternaria*.

On the other hand, species-specific molecular genetic assays are suitable for differentiation between different taxa, depending on the specificity of the respective assay. However, our data and various tests of DNA extraction protocols show that there is a lack of quantitative sample processing and DNA extraction, making molecular genetic quantification inferior to other tools. Nevertheless, molecular genetic tools are still an advancing field in environmental research, where their strengths and limitations are being tested, and advancing technologies such as third-generation sequencing or digital PCR are being established. Therefore, the limits experienced in this study regarding quantification may be overcome with new emerging technologies and optimized protocols.

Although the tested methods show mostly good correlations regarding their quantitative results, even our extensive tests under controlled environments do not allow transposition of one type of result to another, for example, estimating the antigen content based on the spore count. Therefore, there is still a gap in the lack of coupled analysis, using culture characteristics, genomic, and proteomic approaches. The combination of different strategies in field sample analyses can help characterize different properties of the bioaerosol and provide valuable information for analysis (48).

As there is still no dose-response relationship, no guidelines for indoor, outdoor, and occupational environment fungal concentration limits exist, while many questions remain open regarding parameters of microbial air quality, detailed and comprehensive studies are needed to assess existing exposures in relation to health outcomes. Immunological methods such as the ELISAs validated in this study could make an important contribution.

## SUPPLEMENTAL MATERIAL

Supplemental material aem.00163-25-s0001.pdf Figures S1 to S4 and Table S1.

ASM does not own the copyrights to Supplemental Material that may be linked to, or accessed through, an article. The authors have granted ASM a non-exclusive, world-wide license to publish the Supplemental Material files. Please contact the corresponding author directly for reuse.
