# Erratum for Pogner et al., “Quantification of airborne fungal antigens by ELISA and comparison to molecular biological and classical methods”

**DOI:** 10.1128/aem.02189-25

**Published:** 2025-11-21

**Authors:** C.-E. Pogner, M. Gorfer, M. Raulf, J. Strauss, I. Sander

## ERRATUM

Volume 91, no. 8, e00163-25, 2025, https://doi.org/10.1128/aem.00163-25. Page 1, abstract, line 12: “103 to 104 spores/mL” should read “10^3^ to 10^4^ spores/mL.”

