# Supplementary material for: Quantification of airborne fungal antigens by ELISA and comparison to molecular biological and classical methods
Source: Appl Environ Microbiol. 2025 Jul 16;91(8):e00163-25. doi: 10.1128/aem.00163-25 (PMC12366363; doi:10.1128/aem.00163-25)
Supplement: Supplemental material — Figures S1 to S4 and Table S1. [file aem.00163-25-s0001.pdf]

## Supplementary

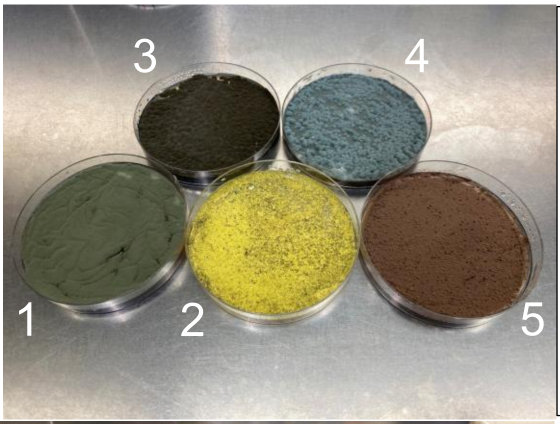


1. *Aspergillus amoenus*
2. *Aspergillus amstelodami*
3. *Cladosporium herbarum*
4. *Penicillium chrysogenum*
5. *Wallemia sebi*

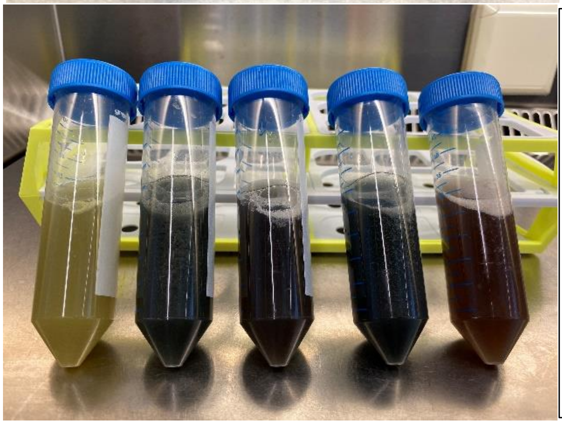

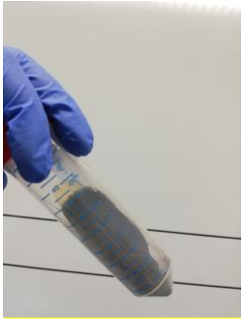

*Aspergillus fumigatus*

*Supp. Figure 1 – Fungal species used for aerosol production; top – fungal species grown in petri dish, below – A. fumigatus grown in 50 mL reaction tubes filled with angular agar*

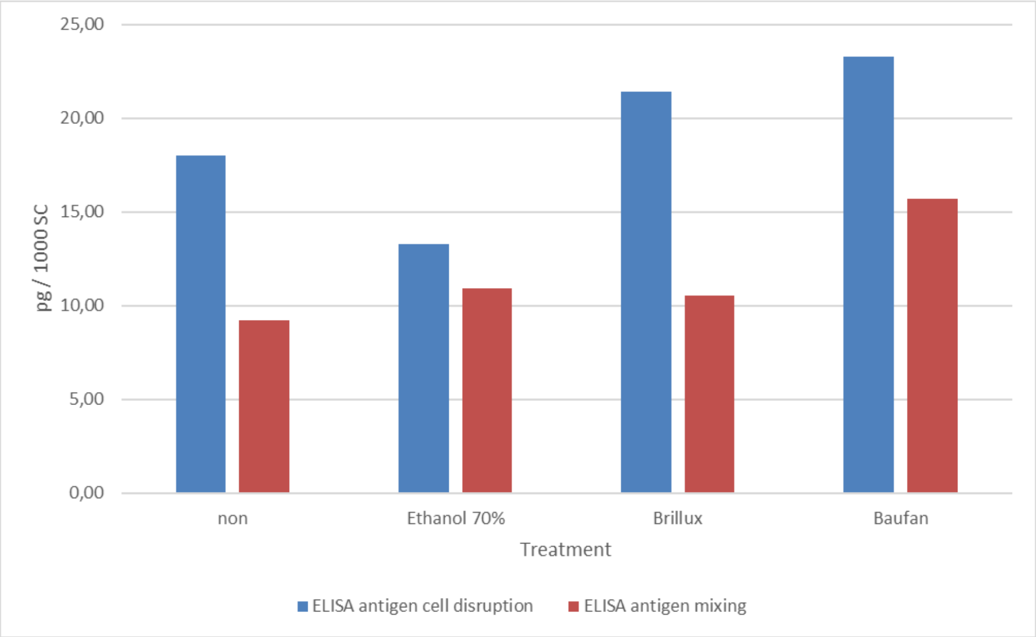


*Supp. Figure 2 – Difference in ELISA recognition for Aspergillus fumigatus spores treated and untreated with fungicide, 5.31x10$^{7}$ spores / mL. Ethanol – adding 99% ethanol to achieve a 70% concentration in the spore suspension; Brillux – using a 1:2 dilution, Baufan – using a 1:40 dilution, adding 0.5 mL each to 3 mL spore suspension.*

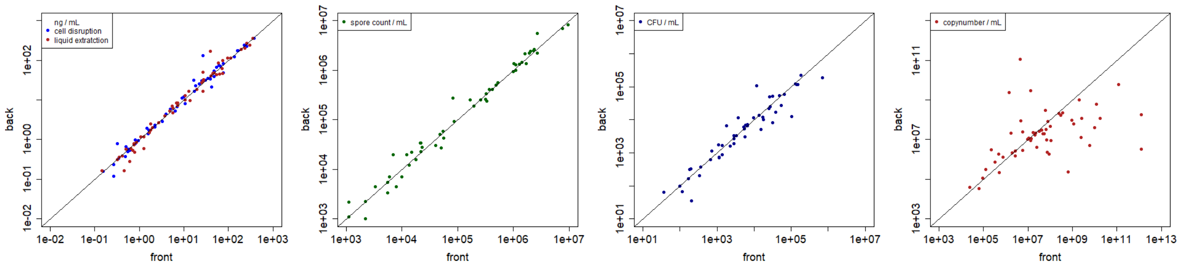


*Supp. Figure 3– Results of sampling pairs (front, back) of the different detection methods; line – fictitious perfect correlation of the sampling pairs; red – ELISA with liquid extraction (rho = 0.986; n = 117); blue – ELISA with cell disruption (rho = 0.983; n = 111); green – spore counting (rho = 0.989; n = 94); blue - CFU (rho = 0.882; n = 99); red - copynumber (rho = 0.578; n = 111); the number of used samples deviates as not all samples were above the detection limit for all methods*

*Supp. Table 1– Spearman correlation coefficient (ρ) for air samples derived from starting material of a mixture of spores from five fungal species (A. amoenus, A. amstelodami, C. herbarum, P. chrysogenum, W. sebi), comparing the detection methods. grey - ρ ≥ 0.75; bold - ρ ≥ 0.9; cd – cell disruption, mix –extraction by mixing*

| *A.amoenus* | CFU | qPCR | ELISA LE | ELISA DC |
|---|---|---|---|---|
| SC | | | | |
| CFU | | -0,661 | -0,674 | -0,676 |
| qPCR | | | 0,885 | **0,908** |
| *A.amstelodami* | CFU | qPCR | ELISA LE | ELISA DC |
| SC | -0,009 | 0,086 | -0,067 | -0,115 |
| CFU | | 0,873 | **0,909** | **0,936** |
| qPCR | | | 0,81 | 0,835 |
| *C.herbarum* | CFU | qPCR | ELISA LE | ELISA DC |
| SC | | 0,552 | 0,617 | 0,539 |
| CFU | | | -0,661 | -0,661 |
| qPCR | | | 0,732 | 0,775 |
| *P.chrysogenum* | CFU | qPCR | ELISA LE | ELISA DC |
| SC | | | | |
| CFU | | 0,77 | **0,955** | **0,936** |
| qPCR | | | 0,848 | 0,818 |
| *W.sebi* | CFU | qPCR | ELISA LE | ELISA DC |
| SC | | | | |
| CFU | | 0,855 | 0,891 | 0,883 |
| qPCR | | | 0,886 | 0,825 |

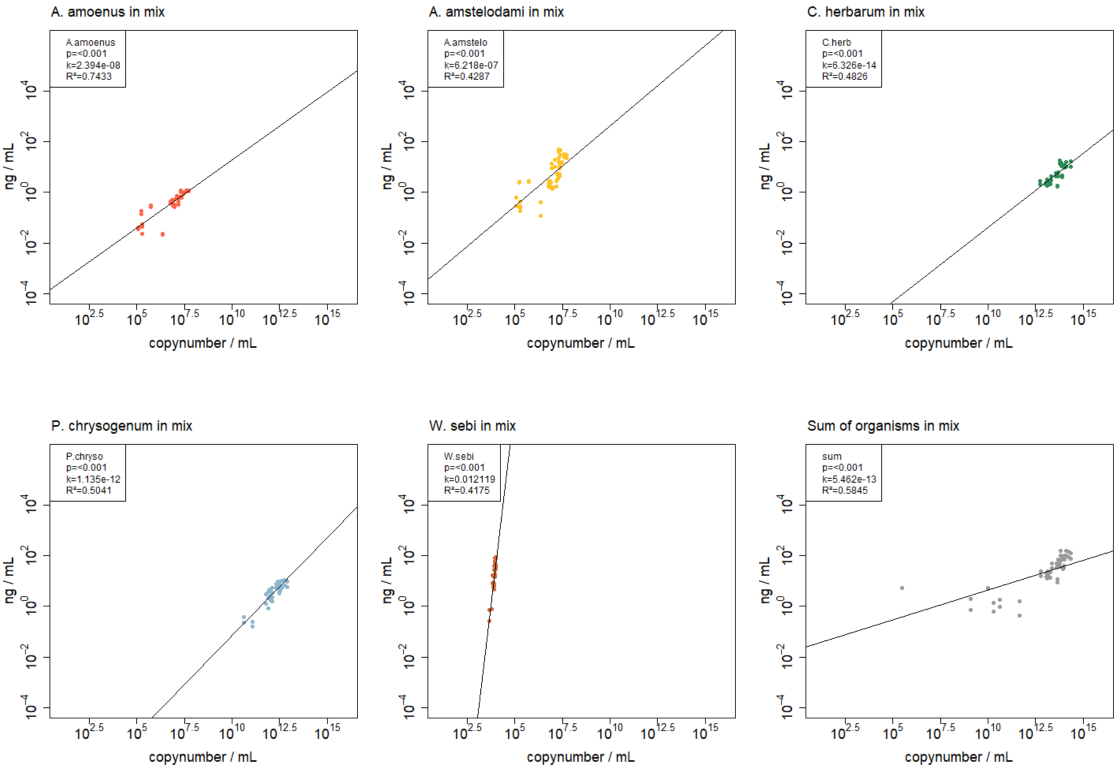


*Supp. Figure 4 – Results for linear correlation of copynumbers / mL and antigen / mL of air samples derived from starting material of a mix of spores from five fungal species (A. amoenus, A. amstelodami, C. herbarum, P. chrysogenum, W. sebi)*
